# Associations of body mass index and metabolic health with stroke risk in a large prospective cohort with time updated covariates

**DOI:** 10.1038/s41598-026-55119-2

**Published:** 2026-05-28

**Authors:** Oskar Hultstrand, Anton Jernberg, David Darehed, Eva-Lotta Glader, Marie Eriksson

**Affiliations:** 1 Department of Statistics, USBE, Umeå University, Umeå, SE-901 87 Sweden; 2 Department of Public Health and Clinical Medicine, Sunderby Research Unit, Umeå University, Umeå, Sweden; 3 Department of Public Health and Clinical Medicine, Umeå University, Umeå, Sweden

**Keywords:** Stroke risk, Obesity, Body mass index, Metabolic syndrome, Survival analysis, Machine learning, Diseases, Health care, Medical research, Neurology, Risk factors

## Abstract

The global prevalence of overweight and obesity is rising, and recent studies have established an independent contribution of adiposity to stroke risk. How the increased risk associated with adiposity relates to other factors including metabolic health is not fully understood. We analyzed 132,045 participants from the Northern Sweden Health and Disease Study with repeated health examinations (1985–2022). Stroke events were identified via national registers. Body mass index (BMI) was modeled as continuous (splines) and categorical (WHO definitions). Cox models with time-updated covariates estimated hazard ratios (HRs), and machine learning (XGBoost-AFT) assessed complex relationships. Over a median 20.2-year follow-up (2.67 million person-years), 7,493 strokes occurred. In fully adjusted models, overweight (HR: 1.14; 95% CI: 1.08–1.20) and obesity (HR: 1.36; 95% CI: 1.27–1.45) independently increased stroke risk versus normal weight. Poor metabolic health was also strongly associated (HR: 1.41 ; 95% CI: 1.34–1.49) with increased stroke risk. Combined obesity and poor metabolic health conferred the highest risk (HR: 1.79; 95% CI: 1.67–1.93). Age modified the association between higher BMI and stroke risk (p-interaction = 0.007), with stronger associations at younger ages. Machine learning confirmed the BMI-stroke risk pattern. In conclusion, overweight and obesity are associated with an independent stroke risk, even among younger and metabolically healthy individuals.

**Supplementary Information:**

The online version contains supplementary material available at 10.1038/s41598-026-55119-2.

## Introduction

There is a rapidly increasing prevalence of overweight and obesity worldwide, and this trend is expected to continue^1^. Obesity contributes to major cardiovascular risk factors including hypertension, dyslipidemia, and type 2 diabetes, and may also increase the risk of stroke and other cardiovascular diseases through chronic inflammation, endothelial dysfunction, pro-thrombotic pathways, carotid atherosclerosis, and a higher prevalence of atrial fibrillation^2–4^. Globally, high body mass index (BMI) accounted for approximately 172,000 ischemic stroke deaths and 4.4 million disability-adjusted life years in 2021, representing a 95% increase in deaths since 1990^5,6^.

According to the World Health Organization (WHO), BMI ≥ 25 kg/m² is classified as overweight and ≥ 30 kg/m² as obesity^7^. Although BMI can be criticized for not distinguishing between fat and lean mass or accounting for fat distribution, it is widely used in epidemiological studies and clinical practice due to its simplicity and strong correlation with health outcomes. While the association between excess weight and stroke risk is well established, evidence on whether overweight and obesity are independent risk factors remains heterogeneous, raising the question about metabolically healthy overweight and obesity. A pooled analysis of 97 prospective cohorts suggested that overweight was not an independent risk factor, whereas approximately one-quarter of obesity’s effect on stroke was not mediated through metabolic factors such as blood pressure, glucose, and cholesterol^8^. Another meta-analysis reported similar results, where metabolically healthy obese individuals had an increased risk of stroke compared to metabolically healthy normal-weight and overweight individuals^9^. In contrast, recent findings from the Norwegian HUNT Study did not demonstrate an independent effect of obesity in metabolically healthy individuals^10^. One possible reason for the diverging results is that the independent effect of overweight and obesity varies by sex and age^11,12^. Moreover, metabolically healthy obesity appears to be a transient state linked to future metabolic deterioration, suggesting a temporal dimension to risk^13^. These findings highlight the need for longitudinal studies that account for sex, age, and changes in metabolic health over time.

Clarifying these relationships is essential for risk stratification and prevention strategies, given the growing global burden of stroke attributable to obesity. To address these gaps, we investigated the association between BMI, metabolic health, sex, age, and stroke incidence in a large Swedish cohort with long-term follow-up and repeated measurements of cardiovascular risk factors using advanced statistical methods. Specifically, we aimed to (1) evaluate stroke risk across BMI categories, with particular focus on individuals with obesity; (2) determine whether obesity is associated with an excess risk independent of metabolic health status, and how the relationship relates to sex, age, and other factors. By modeling BMI as a time-updated exposure and applying both traditional and machine learning-based survival models, we sought to provide a comprehensive and temporally dynamic analysis of stroke risk.

## Methods

### Material

This study was based on data from the NSHDS, a large population-based longitudinal cohort comprising three health examination programs in the two northernmost regions in Sweden: the Västerbotten Intervention Programme (VIP), the Northern Sweden Monitoring of Trends and Determinants of Cardiovascular Disease (MONICA) Study, and the Mammography Screening Project^14^. We included participants who underwent at least one health examination between 1985 and 2024 in VIP or MONICA. Each examination collected information on anthropometric measures, blood pressure, blood lipids, and glucose tolerance.

Stroke events were identified through linkage with Riksstroke^15^ and the Swedish national patient register managed by the National Board of Health and Welfare^16^. Participants were followed from the date of their first health examination until stroke diagnosis, death, or administrative censoring on June 30, 2024, whichever occurred first.

### Variable definitions

The outcome of interest included ischemic strokes and intracerebral hemorrhages (International Classification of Disease, ICD-9 codes: 431, 433, 434, 436, and ICD-10 codes: I61, I63, I64).

The main exposure, BMI, was calculated as weight (kg) divided by height (m²) and modeled both continuously and categorically using the WHO definitions^7^: underweight (< 18.5), normal weight (18.5–24.9), overweight (25–29.9), and obesity (≥ 30).

Metabolic health was defined as the absence of the following risk factors: systolic blood pressure > 140 mmHg, diastolic blood pressure > 90 mmHg, fasting glucose > 7 mmol/L, 2-hour post-load glucose > 11 mmol/L, total cholesterol > 6.2 mmol/L, or physician-diagnosed diabetes. Poor metabolic health was defined as having at least one of these risk factors. A four-level composite variable combining obesity (yes/no) and metabolic health (good/poor) was constructed to explore interactive and combined effects.

Other covariates included age (years), sex, smoking status (current, former, never), highest attained education (primary school, secondary school, university), and calendar decade of health examination. All variables were updated at each subsequent health examination.

### Statistical methods

We used Cox proportional hazards models with time-updated covariates, applying a counting process formulation^17^. Each individual contributed one or more observation intervals during which covariates were assumed constant. Hazard ratios (HRs) and 95% confidence intervals (CIs) were estimated with robust standard errors to account for intra-individual correlation due to repeated measures. Non-linear associations were examined using natural splines with model selection guided by Akaike Information Criterion (AIC).

Effect modification was assessed by testing interaction terms between BMI and other covariates using likelihood ratio tests. We examined the log(-log) survival plots to detect major violations from the proportional hazard assumption.

Covariates were added to the Cox regression model sequentially. A univariable model including BMI alone (Model 1), and multivariable models including BMI + metabolic health (Model 2), and BMI + metabolic health + smoking, age, sex, education, and decade of health examination (Model 3). We used a complete case approach, excluding observations with missing information.

To evaluate the robustness of our findings, we conducted sensitivity analyses by censoring individuals 15 years after their last health examination.

To capture potential non-linear and complex relationships between covariates and risk of stroke, we additionally applied a machine learning approach. A gradient-boosted Accelerated Failure Time (XGBoost-AFT) model^18^ was trained using 80% of the dataset for model development and 20% for validation. Hyperparameters were tuned via grid search with cross-validation (Supplementary table S1). Model performance was evaluated using the concordance index (C-index). To enhance interpretability, SHapley Additive exPlanations (SHAP) values^19^ were computed to quantify the contribution of each covariate to predicted stroke survival times.

The main statistical analyses, including Cox regression, were performed in R (version 4.5.0; R Core Team, 2025) using the survival package. Machine learning was performed in Python (version 3.11.12; Python Software Foundation, 2025) using packages sklearn.model_selection, xgboost and shap.

### Ethical considerations

The study was conducted in accordance with the Declaration of Helsinki and approved by the Swedish Ethical Review Authority (ref no. 2023-07750-01). All participants in NSHDS provided informed consent for their data to be used for research purposes. Data was pseudonymized before researcher access.

The reporting of this study complies with the STrengthening the Reporting of Observational studies in Epidemiology (STROBE) guideline.

## Results

### Study population

There were 135,124 individuals participating in 214,781 health examinations 1985–2022. Information on one or more key variables was missing on 5836 (2.7%) of health examinations (BMI was missing in 1660 examinations) and these were excluded from analyses. The final analytic cohort comprised 208,945 health examinations in 132,045 individuals (74,115 participated in one, 40,601 in two, and 17,329 in three or more health examinations). The total follow-up time was 2,672,282 person-years, with a median follow-up of 20.2 years (IQR, 13.8–26.7). During follow-up, 7,493 participants experienced a stroke of which 985 (13.1%) were intracerebral hemorrhages, 18,861 died without prior stroke, and 105,691 were censored at the end of follow-up (June 30, 2024).

Baseline characteristics are presented in Table 1. At the first health examination, 65,278 (49.4%) were men, the mean age was 46.3 years, mean BMI 26.0 kg/m², and 38.7% had poor metabolic health. The BMI distribution was 45.0% normal weight, 38.5% overweight, and 15.7% obese. The underweight group was small in comparison (0.8%). The proportions of men, former smokers, participants with poor metabolic health, and lower education, were higher in the overweight and obesity groups compared with the normal weight group (Table 2).

**Table 1:** Baseline characteristics of participants at first health examination and at all examinations. Frequency (n) and proportions (%).

Variable	At first examination (*N* = 132,045)	All examinations (*N* = 208,945)
Men (n, %)	65 278 (49.4%)	101 672 (48.7%)
Age (years, mean)	46.3	49.1
Poor metabolic health (n, %)	51 123 (38.7%)	85 772 (41.1%)
BMI (kg/m^2^, mean)	26.0	26.3
BMI class (n, %)		
Underweight	1 036 (0.8%)	1 433 (0.7%)
Normal weight	59 455 (45.0%)	88 579 (42.4%)
Overweight	50 827 (38.5%)	83 448 (39.9%)
Obesity	20 727 (15.7%)	35 485 (17.0%)
Smoking status (n, %)		
Never smoker	83 525 (63.3%)	133 719 (64.0%)
Former smoker	26 542 (20.1%)	44 418 (21.3%)
Current smoker	21 978 (16.6%)	30 808 (14.7%)
Education (n, %)		
Primary school	26 966 (20.4%)	39 056 (18.7%)
Secondary school	65 953 (49.9%)	107 437 (51.4%)
University	39 126 (29.6%)	62 452 (29.9%)
Decade of examination (n, %)		
1980s	4 577 (3.5%)	4 595 (2.2%)
1990s	63 401 (48.0%)	69 516 (33.3%)
2000s	33 018 (25.0%)	67 281 (32.2%)
2010s	28 761 (21.8%)	62 814 (30.1%)
2020s	2 288 (1.7%)	4 739 (2.3%)

**Table 2:** Descriptive statistics of health examination participants in each BMI category (all health examinations).

Variable	Underweight (*N* = 1433)	Normal weight (*N* = 88579)	Overweight (*N* = 83448)	Obese (*N* = 35485)
BMI (mean, sd)	17.68 (0.72)	22.68 (1.55)	27.10 (1.38)	33.61 (3.75)
Age (mean, sd)	46.37 (10.44)	47.46 (9.61)	50.15 (9.20)	50.64 (9.06)
Men (n, %)	262 (18.3%)	35,493 (40.1%)	48,628 (58.3%)	17,289 (48.7%)
Poor metabolic health (n, %)	323 (22.5%)	26,793 (30.2%)	38,380 (46.0%)	20,276 (57.1%)
Smoking status (n, %)				
Never smoker	867 (60.5%)	58,629 (66.2%)	52,705 (63.2%)	21,518 (60.6%)
Former smoker	181 (12.6%)	15,881 (17.9%)	19,208 (23.0%)	9148 (25.8%)
Current smoker	385 (26.9%)	14,069 (15.9%)	11,535 (13.8%)	4819 (13.6%)
Education (n, %)				
Primary school	231 (16.1%)	13,955 (15.8%)	16,926 (20.3%)	7944 (22.4%)
Secondary school	672 (46.9%)	43,204 (48.8%)	44,025 (52.8%)	19,536 (55.1%)
University	530 (37.0%)	31,420 (35.5%)	22,497 (27.0%)	8005 (22.6%)
Decade of examination (n, %)				
1980s	45 (3.1%)	2513 (2.8%)	1582 (1.9%)	455 (1.3%)
1990s	505 (35.2%)	33,177 (37.5%)	27,226 (32.6%)	8608 (24.3%)
2000s	429 (29.9%)	27,538 (31.1%)	27,739 (33.2%)	11,575 (32.6%)
2010s	428 (29.9%)	23,686 (26.7%)	25,057 (30.0%)	13,643 (38.4%)
2020s	26 (1.8%)	1665 (1.9%)	1844 (2.2%)	1204 (3.4%)

### Risk of stroke

#### Unadjusted analysis of BMI and stroke risk

**Fig. 1:**
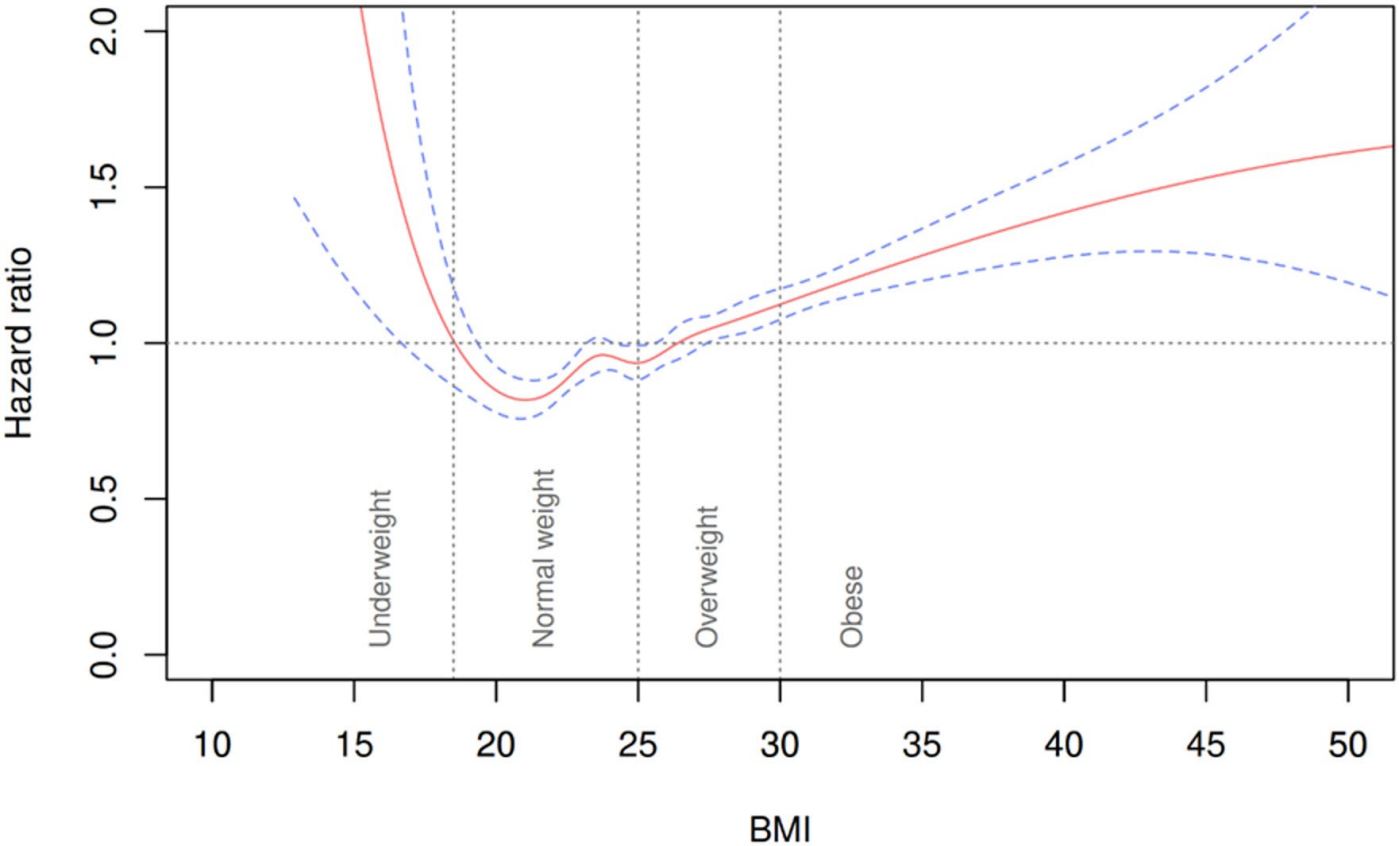
Cox proportional hazard regression modelling the non-linear association between BMI (continuous) and time to stroke. Vertical dotted lines represent the WHO cutoffs for BMI categories underweight, normal weight, overweight and obesity. Estimated hazard ratio (red line) with 95% confidence intervals (blue dashed lines).

Modeling BMI as a continuous variable using natural splines revealed a U-shaped association with stroke risk (Fig. 1). The lowest estimated hazard occurred at BMI 21–22 kg/m² with hazard increasing below 20 kg/m² and above 25 kg/m². This pattern supported the use of WHO BMI categories for risk stratification.

In univariable Cox regression (model 1), both overweight (HR: 1.42, 95% CI: 1.35–1.49) and obesity (HR: 1.57, 95% CI: 1.47–1.67) were associated with an increased stroke risk compared to normal weight (Table 3).

**Table 3:** Cox proportional hazard regression modelling the association between BMI and time to stroke.

Variable	Model 1	Model 2	Model 3
BMI class			
Underweight	1.25 (0.94, 1.67)	1.37 (1.03, 1.83)	1.53 (1.14, 2.04)
Normal weight	1.00 (ref)	1.00 (ref)	1.00 (ref)
Overweight	1.42 (1.35, 1.49)	1.25 (1.19, 1.32)	1.14 (1.08, 1.20)
Obesity	1.57 (1.47, 1.67)	1.28 (1.20, 1.37)	1.36 (1.27, 1.45)
Metabolic health			
Healthy	1.00 (ref)	1.00 (ref)	1.00 (ref)
Unhealthy	2.40 (2.28, 2.52)	2.30 (2.19, 2.42)	1.41 (1.34, 1.49)
Smoking status			
Never smoker	1.00 (ref)	--	1.00 (ref)
Former smoker	1.41 (1.34, 1.49)	--	1.09 (1.03, 1.15)
Current smoker	1.68 (1.58, 1.78)	--	1.53 (1.45, 1.63)
Age	Nonlinear (natural spline)	--	Nonlinear (natural spline)
Sex			
Female	1.00 (ref)	--	1.00 (ref)
Male	1.50 (1.43, 1.57)	--	1.58 (1.51, 1.65)
Education level			
Primary school	1.00 (ref)	--	1.00 (ref)
Secondary school	0.49 (0.47, 0.52)	--	0.95 (0.90, 1.00)
University	0.36 (0.33, 0.38)	--	0.85 (0.79, 0.91)
Decade of examination			
1980s	1.00 (ref)	--	1.00 (ref)
1990s	1.20 (1.05, 1.37)	--	0.90 (0.79, 1.03)
2000s	0.70 (0.61, 0.81)	--	0.51 (0.44, 0.58)
2010s	0.32 (0.24, 0.58)	--	0.24 (0.21, 0.28)
2020s	0.37 (0.24, 0.58)	--	0.25 (0.16, 0.38)

Separate univariable models for BMI and other covariates (Model 1), and multivariable models adjusting for metabolic health (Model 2), and further adjusting for smoking, age, sex, education, calendar decade (Model 3). Age was modeled using a natural cubic spline with 5 degrees of freedom, with median age as reference. Hazard Ratios (95% confidence intervals).

#### Adjusted analysis of BMI and stroke risk

Including BMI and metabolic health in the same multivariable model (model 2), showed that overweight (HR: 1.25, 95% CI: 1.19–1.32), obesity (HR: 1.28, 95% CI: 1.20–1.37), and poor metabolic health (HR: 2.30, 95% CI: 2.19–2.42) were independently associated with an increased risk of stroke (Table 3).

Age strongly influenced stroke risk, each 10-year increase on average doubled the risk (HR: 2.22, 95% CI: 2.15–2.30). Natural spline modeling demonstrated a nonlinear association with stroke risk (Figure S1). The hazard ratio remained relatively unchanged from early adulthood until approximately 50 years of age, after which it increased progressively, indicating an accelerating risk of stroke with advancing age. Age was therefore modelled as a spline in the fully adjusted models. After adjusting for metabolic health, age, sex, education level, smoking status, and examination decade, the increased risk of stroke associated with overweight, and obesity compared to normal weight was reduced but remained significant (HR: 1.14, 95% CI: 1.08, 1.20, and HR: 1.36, 95% CI: 1.27–1.45 respectively). Underweight was also associated with an elevated risk (HR 1.53; 95% CI, 1.14–2.04), though estimates were less precise due to small numbers.

### Effect modification

Adding an age-by-BMI interaction to the fully adjusted model (model 3) revealed that obesity-related stroke risk declined with age (p for interaction = 0.007). HR for obesity was 1.96 at age 30, decreasing to 1.00 at age 80 compared to normal weight. No significant interactions were observed between BMI and sex, metabolic health, smoking, education, or calendar period.

### Combined effects of obesity and metabolic health

The non-significant interaction between BMI and metabolic health suggested a multiplicative effect on stroke risk, i.e. that overweight and obesity increase the risk by a similar factor in individuals with poor and good metabolic health. Compared to metabolically healthy non-obese individuals, those with both obesity and poor metabolic health had the highest risk (HR 1.79; 95% CI, 1.67–1.93). Elevated risks were also observed for non-obese individuals with poor metabolic health (HR 1.43; 95% CI, 1.35–1.51) and metabolically healthy individuals with obesity (HR 1.25; 95% CI, 1.12–1.40). These findings indicate that obesity and metabolic dysfunction independently contribute to stroke risk, with the greatest burden in individuals with both factors.

### Proportional hazard assumption and sensitivity analyses

Log(-log) survival plots showed no major violations of proportional hazard assumption.

We assessed exposure misclassification by restricting follow-up to 15 years after last health examination. This did not alter associations; effect sizes were slightly stronger (obesity HR 1.45 (95% CI, 1.34–1.58) and poor metabolic health HR 1.52 (95% CI, 1.42–1.62)). Other covariates showed stable or slightly stronger associations, confirming robustness (supplementary table S3).

### Machine learning results

The XGBoost AFT model achieved a C-index of 0.78 in the validation dataset. Variable importance analysis identified age, calendar decade, and metabolic health as the most influential predictors. BMI contributed meaningfully but had a lower gain per split (Supplementary Table 2).

**Fig. 2:**
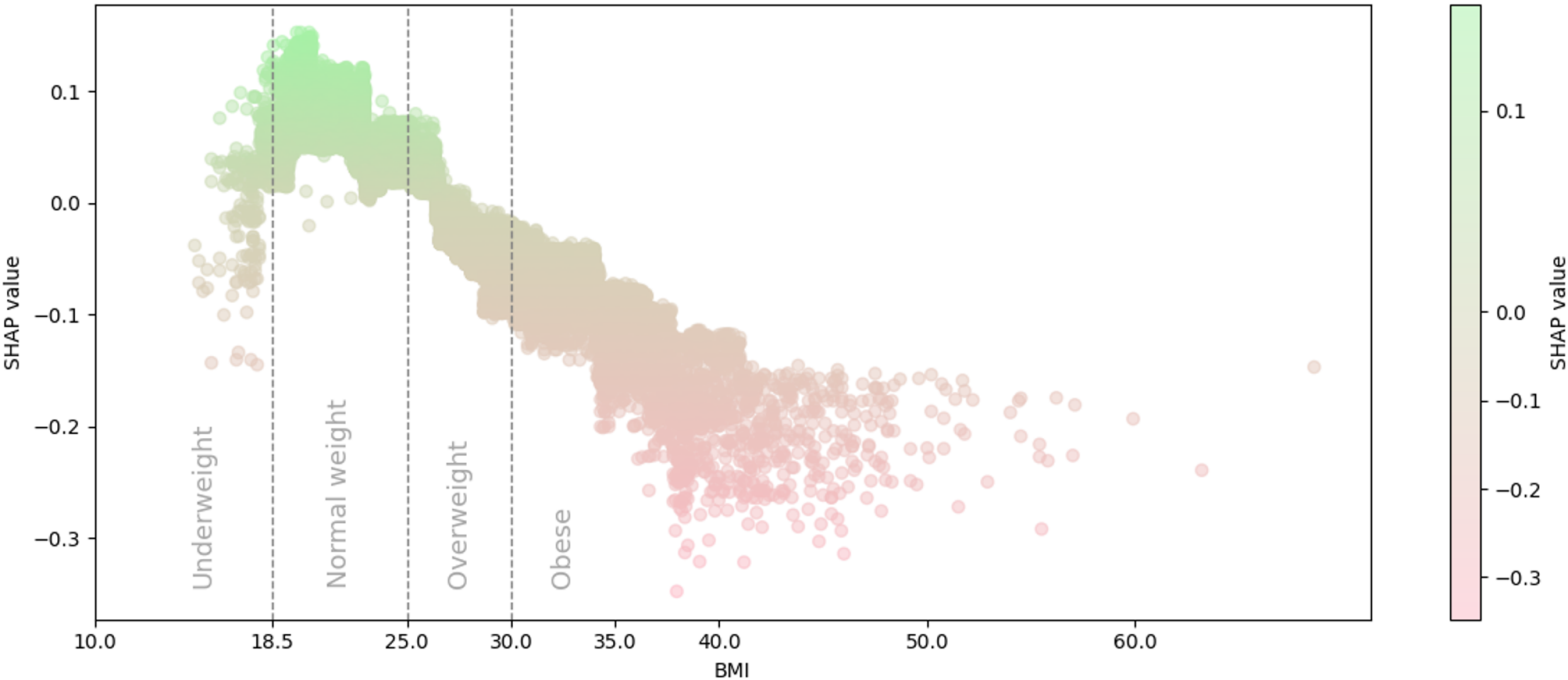
SHAP values illustrating the contribution of BMI to predicted stroke-free survival from the gradient-boosted Accelerated Failure Time (XGBoost-AFT) model. A lower SHAP value (red) is associated with an increased predicted probability of stroke. Vertical dotted lines represent the WHO cutoffs for BMI categories underweight, normal weight, overweight and obesity.

SHAP analysis revealed a non-linear relationship between BMI and stroke (Fig. 2), consistent with the U-shaped pattern from the spline-based Cox model. The strongest negative SHAP values (indicating increased risk of stroke) occurred in participants with obesity, while underweight and overweight showed modest risk elevations.

## Discussion

In this large, population-based cohort with long-term follow-up and repeated measurements, we found that both overweight and, more strongly, obesity was associated with an increased risk of stroke compared to normal weight, even after adjusting for metabolic health and other confounders. Poor metabolic health was a powerful independent predictor of stroke, and individuals with both obesity and poor metabolic health had the highest risk. These findings were consistent across multiple analytical approaches including spline-based modeling, interaction analysis, and machine learning-based survival prediction and provide evidence against the concept of “metabolically healthy obesity” underscoring the dual importance of adiposity and metabolic dysfunction in stroke prevention.

Our results align with previous meta-analyses indicating that a substantial proportion of obesity-related stroke risk is mediated through metabolic factors but not entirely explained by them^8,9^. The observation that metabolically healthy obesity still conferred a modestly elevated risk supports the hypothesis that obesity exerts direct vascular effects beyond traditional risk factors, possibly through inflammatory and pro-thrombotic pathways^4^. Conversely, the strong effect of poor metabolic health among non-obese individuals highlights the need for comprehensive risk assessment beyond BMI alone. Currently, body weight is not included in most structured cardiovascular or stroke risk assessment tools^20^. However^21,22^, as evidence increasingly supports obesity as an independent and modifiable risk factor, systematic weight management should be integrated into stroke prevention strategies. Effective obesity management remains an often underutilized yet essential component of both primary and secondary stroke prevention.

Demographic heterogeneities have been reported for cardiovascular outcomes, suggesting that relative risk associated with obesity may be more pronounced in younger adults and in women^11,12^. Our study found no evidence of sex-related differences in the BMI-stroke association. However, the age interaction observed, where obesity-related stroke risk declined with advancing age, adds nuance to existing literature. This may reflect survival bias or differential physiological resilience with age. Similar trends have been described in studies of the so-called “obesity paradox,” where higher BMI appears protective among older or hospitalized stroke patients^23^. In our analysis of incident stroke, the limited association at older ages likely reflects changing risk profiles rather than causal protection. These findings suggest that early adulthood may represent a critical window for intervention.

The U-shaped association between BMI and stroke risk observed in our spline models supports previous findings that underweight individuals may also face cerebrovascular risk^24,25^. While our primary focus was overweight and obesity, we did not account for potential confounders linked to low body weight, such as frailty and multimorbidity, which have been associated with elevated stroke risk in prior studies^26^.

The major strengths of this study include its prospective design, extensive follow-up, repeated measurements, large sample size, as well as individual linkage to national stroke and mortality registries enabling precise outcome ascertainment. The use of time-updated covariates reduced misclassification and allowed us to capture dynamic risk patterns. Although not all participants had repeated examinations, this approach still reduces exposure misclassification compared with baseline-only analyses, which implicitly assume lifelong stability. The sensitivity analysis, censoring individuals 15 years after their last examination, showed slightly stronger effect estimates, supporting the robustness of our findings and suggesting that long intervals without updated information did not substantially distort the associations. Our complementary machine learning approach allowed flexible modelling of complex relationships and supported findings from the traditional Cox model, enhancing analytical rigor.

Limitations include the observational design and potential residual confounding by unmeasured factors such as genetics and diet. Metabolic health was modeled as a binary variable, which may oversimplify heterogeneous cardiometabolic risk profiles. Individuals with a single mildly elevated risk factor were grouped together with those with multiple concurrent abnormalities. However, there is no universally accepted definition of metabolic health, and our approach was chosen to prioritize transparency and alignment with clinically used thresholds. Future studies could explore graded definitions of metabolic health to capture risk heterogeneity in greater detail. Importantly, despite this simplification, we observed strong and consistent independent effects of both metabolic health and BMI, suggesting that the main conclusions are robust. BMI, while widely used, does not distinguish between fat and lean mass or account for fat distribution, which may better predict stroke risk. NSHDS has a high participation rate (~ 70%), but non-participants tended to be younger and of lower socioeconomic status^14^, which could influence effect estimates. These factors may affect the magnitude of associations but are unlikely to alter overall conclusions.

Second, ischemic stroke and intracerebral hemorrhage were analyzed as a composite outcome. Although these subtypes differ in pathophysiology and risk factor profiles, stratified analyses were not prioritized due to limited precision for intracerebral hemorrhage when accounting for time‑updated BMI and metabolic health. Our findings therefore primarily reflect overall stroke risk, and future studies with sufficient subtype‑specific power should examine potential heterogeneity by stroke subtype.

Given the analytical complexity of multiple imputation for time-updated covariates in a counting-process survival framework, complete case analysis was considered appropriate. While multiple imputation could in principle reduce selection bias, the low proportion of missing data suggests that missingness is unlikely to have any substantial impact on our conclusions.

Finally, our findings pertain to a Swedish population, and many participants underwent their first health examination in the 2000s or earlier. Hence our findings may not fully generalize to other settings.

Our findings emphasize that stroke prevention strategies should not only target individuals with metabolic abnormalities but also those with obesity, even when metabolic health appears favorable. Early intervention in younger adults may be particularly important given the stronger relative risk observed in this group. Public health policies should integrate weight management with metabolic risk screening to reduce the globally growing burden of stroke attributable to obesity.

## Conclusion

High BMI remains a relevant and independent risk factor for stroke, even among individuals with favorable metabolic profiles. These findings challenge the notion that obesity without metabolic complications is clinically benign and underscore the importance of addressing excess weight as part of primary stroke prevention.

## Supplementary Information

Below is the link to the electronic supplementary material.

Supplementary Material 1

## Data Availability

The data that support the findings of this study are available from the NSHDS, Riksstroke, Statistics Sweden, and the National Board of Health and Welfare, but restrictions apply to the availability of these data, which were used under license for the current study, and so are not publicly available. Data are however available from the authors upon reasonable request and with permission of NSHDS, Riksstroke, Statistics Sweden, and the National Board of Health and Welfare.

## Abbreviations

AIC: Akaike information criterion
BMI: Body mass index
C-index: Concordance index
CI: Confidence interval
HR: Hazard ratio
ICD: International classification of diseases
MONICA: Northern Sweden Monitoring of Trends and Determinants of Cardiovascular Disease
NSHDS: Northern Sweden Health and Disease Study
Riksstroke: The Swedish Stroke Register
SHAP: SHapley Additive exPlanations
VIP: Västerbotten Intervention Programme
XGBoost-AFT: eXtreme Gradient Boosting – Accelerated Failure Time model
