# Supplementary material for: Associations of body mass index and metabolic health with stroke risk in a large prospective cohort with time updated covariates
Source: Sci Rep. 2026 May 28;16:16590. doi: 10.1038/s41598-026-55119-2 (PMC13219419; doi:10.1038/s41598-026-55119-2)
Supplement: Supplementary file 1 — Supplementary Material 1 [file 41598_2026_55119_MOESM1_ESM.pdf]

Supplementary information

The association between BMI, metabolic health, and stroke risk. A prospective cohort study with time-updated covariates

Oskar Hultstrand BSc[#1], Anton Jernberg BSc[#1], David Darehed MD PhD[2],
Eva-Lotta Glader MD PhD[3], Marie Eriksson PhD[*1]

***Table S1**. Hyperparameters of XGBoost model*

	Tested values	Selected model
**Hyperparameters**		
n_estimators	25, 50, 100, 300	25
learning_rate	0.01, 0.1, 0.3	0.1
max_depth	1, 3, 5	3
min_child_weight	0.01, 0.1, 1	0.01
**Preset Parameters**		
objective	survival:aft	survival:aft
eval_metric	aft-nloglik	aft-nloglik
aft_loss_distribution	normal	normal
aft_loss_scale	1.0	1.0
verbosity	0	0
**Model Performance**	**C-index**	
Training data	0.7876	
Validation data	0.7765	

***Table S2.** Feature Importance from XGBoost model*

Variable	Weight	Gain	Cover
Age	208	167	31,236
Decade of examination	173	148	28,799
BMI	118	9	7,183
Smoking	68	20	10,760
Sex	66	30	15,763
Metabolic health	42	50	26,570
Education level	19	31	24,961

Table S3. *Sensitivity analysis censoring individuals 15 years after their last health examination. Cox proportional hazard regression modelling the association between BMI and time to stroke. Multivariable model adjusting for metabolic health, smoking, age (spline), sex, education, calendar decade (Model 3). Hazard Ratios (95% confidence intervals)*

**Variable**	**Censored at 15 years HR (95% CI)**
**BMI class**	
Underweight	1.63 (1.15, 2.32)
Normal weight	1.00 (ref)
Overweight	1.17 (1.09, 1.25)
Obesity	1.45 (1.34, 1.58)
**Metabolic health**	
Healthy	1.00 (ref)
Unhealthy	1.52 (1.42, 1.62)
**Smoking status**	
Never smoker	1.00 (ref)
Former smoker	1.10 (1.02, 1.18)
Current smoker	1.59 (1.48, 1.72)
**Age**	-
**Sex**	
WomanFemale	1.00 (ref)
Male	1.72 (1.62, 1.83)
**Education level**	
Primary school	1.00 (ref)
Secondary school	0.91 (0.85, 0.97)
University	0.82 (0.75, 0.89)
**Decade of examination**	
1980s	1.00 (ref)
1990s	1.02 (0.83, 1.25)
2000s	0.80 (0.65, 0.99)
2010s	0.49 (0.39, 0.61)
2020s	0.45 (0.28, 0.74)

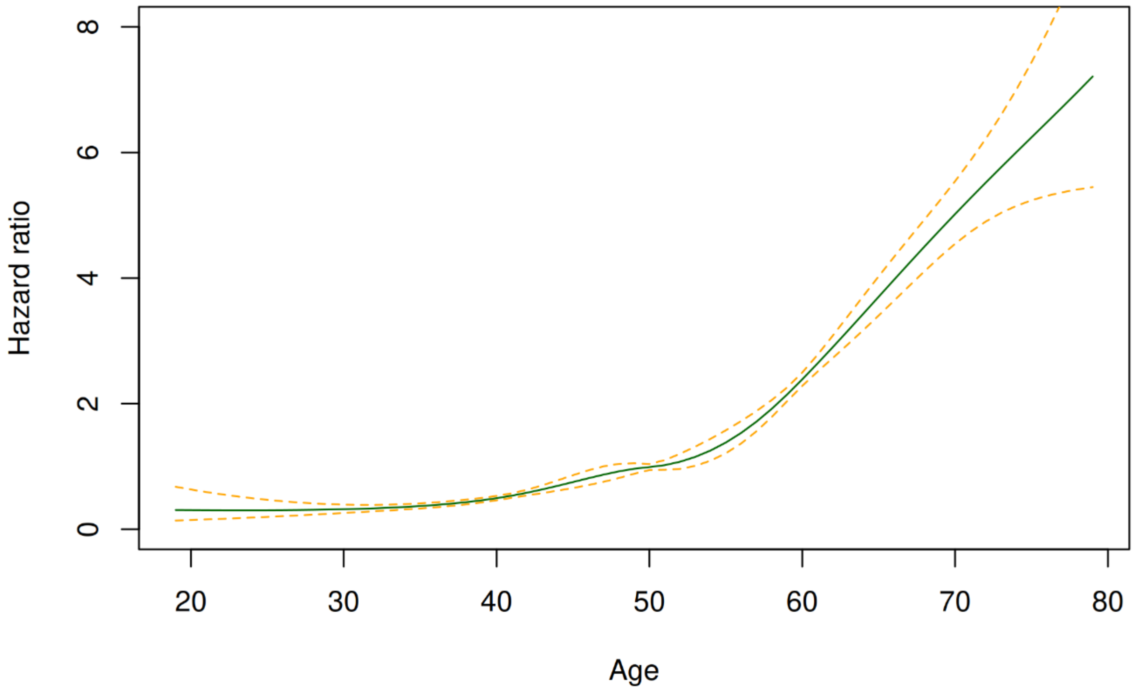

Figure S1. *A natural cubic spline with 5 knots modelling the non-linear effect of age on stroke risk. Hazard ratio relative median age (50 years)*

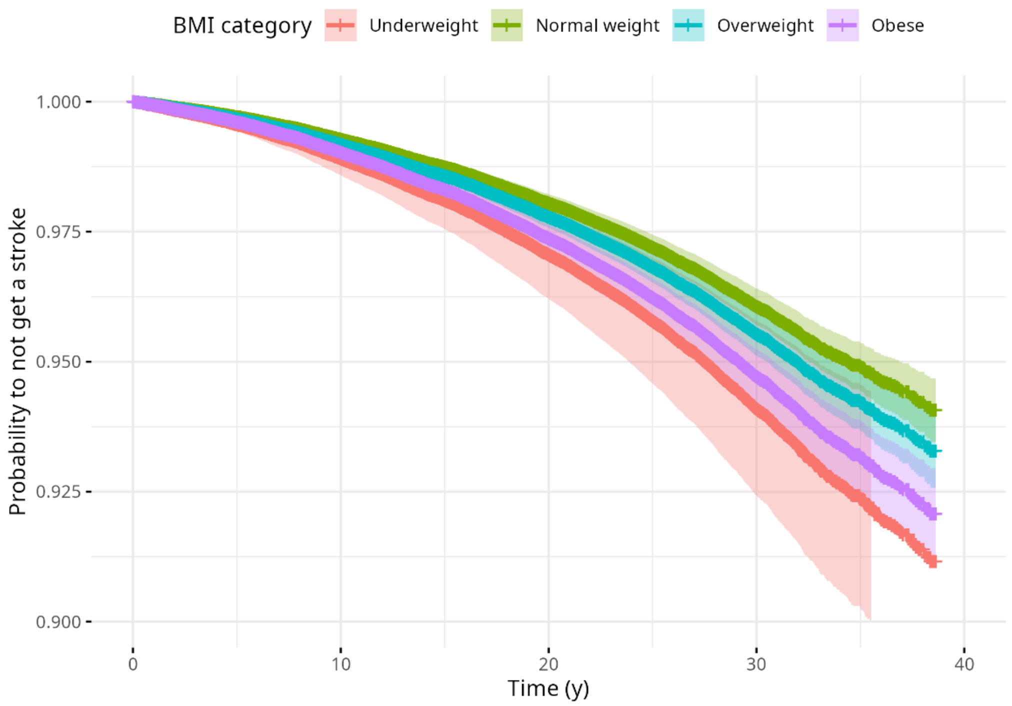

Figure S2. *Estimated probability of stroke-free survival estimated by fully adjusted Cox regression (model 3),evaluated at median age (50 years), examination decade at 2020, and all other covariates held at their reference levels.*
